# Arbuscular mycorrhizal fungi strongly influence the endorhizosphere of grapevine rootstock with soil type as a key factor

**DOI:** 10.1007/s00572-025-01194-8

**Published:** 2025-03-05

**Authors:** K. Štůsková, A. Vavřiník, E. Hakalová, J. Čechová, D. Gramaje, A. Eichmeier

**Affiliations:** 1 Mendeleum Institute of Genetics, Mendel University in Brno, Valtická 334, 69144 Lednice, Czech Republic; 2 Department of Viticulture and Enology, Mendel University in Brno, Valtická 337, 691 44 Lednice, Czech Republic; 3 Instituto de Ciencias de La Vid y del Vino (ICVV), Consejo Superior de Investigaciones Científicas - Universidad de La Rioja - Gobierno de La Rioja, Ctra. LO-20 Salida 13, Finca La Grajera, 26071 Logroño, Spain

**Keywords:** Arbuscular mycorrhizal fungi, Grapevine, Metagenomics, Soil characteristics, Endorhizosphere, Microbiome

## Abstract

Arbuscular mycorrhizal fungi (AMF) play a crucial role in enhancing the health and productivity of host plants, including grapevine. By forming symbiotic relationships with plant roots, AMF significantly improve water uptake and nutrient absorption, particularly phosphorus (P) and nitrogen (N). This study evaluated the microbiome composition and AMF colonization in the grapevine endorhizosphere across five wine-growing sub-regions in the Czech Republic. In all five sub-regions, in terms of composition of the fungal microbiome, the phyla *Ascomycetes* and *Basidiomycetes* were most numerous. Additionally, the study confirmed that LSU primers are more sensitive than ITS primers for AMF sequencing. While the representation of the phylum *Glomeromycetes* ranged from 0.07% to 5.65% in the ITS library, it was significantly higher, ranging from 83.74% to 98.71%, in the LSU library. The most significant difference compared to other sub-regions was observed in the Slovácko sub-region, where the soil had a low pH, a different texture (sandy loam), reduced micronutrient concentration, and low organic matter. The application of chemical plant protection products to grapevines also could have played a significant role, with 49 applications recorded in the Slovácko sub-region during the three years preceding sample collection. In other sub-regions, chemical treatments were conducted only 19–26 times. These factors resulted in only trace amounts of AMF being detected in Slovácko. Furthermore, it was demonstrated that AMF positively influenced the phosphorus concentration in the soil and reduced the presence of certain fungal pathogens.

**Supplementary Information:**

The online version contains supplementary material available at 10.1007/s00572-025-01194-8.

## Introduction

Arbuscular mycorrhizal fungi (AMF) are widespread root symbionts, present in over 90% of vascular plants (Wang and Qiu 2006). Mutual interactions between plants and microbes offer a promising approach to increase crop yields while reducing costs associated with the excessive use of chemical fertilizers. In this context, AMF play a crucial role as soil microorganisms by increasing the interface between roots and soil, thereby enhancing plant nutritional status, particularly phosphorus uptake (Gilbert et al. 2014; Moukarzel et al. 2023). Additionally, AMF provide plants with further benefits, helping them to better tolerate both biotic and abiotic stresses (Bona et al. 2011), as well as improving fruit yield and quality (Todeschini et al. 2018). Secondary benefits of AMF may include reducing infections by root pathogens (Popescu 2016), improving plant water balance (Augé 2001), and decreasing the uptake of heavy metals by plants (Göhre and Paszkowski 2006). However, a reduction in AMF biodiversity can negatively impact plant functionality (Gilbert et al. 2014). The composition of AMF communities might shift according to the plant’s phenological stage, particularly during flowering and maturation, when changes in the composition of root exudates occur (García et al. 2001).

Arbuscular mycorrhiza is the most widespread type of mycorrhiza, and grapevines are particularly sensitive to its presence. AMF have been found to offer protection against several fungal pathogens associated with grapevines (Nogales et al. 2009). Specifically, AMF have been demonstrated to reduce infection and mitigate the effects of grapevine trunk diseases, particularly black foot disease. One study revealed that grapevine rootstock (*'V. rupestris'* Scheele) inoculated with *Rhizophagus intraradices* N.C.Schenck & G.S.Sm. before being exposed to *'Cylindrocarpon' macrodidymum* Schroers, Halleen & Crous (now classified as part of the *Dactylonectria macrodidyma* (Halleen, Schroers & Crous) L.Lombard & Crous species complex) was less susceptible to black foot disease than non-mycorrhizal plants (Petit et al. 2011). Furthermore, recent research suggests that the genetic makeup of host plant species can influence AMF community composition in grapevines, with different rootstocks in the same location hosting different AMF communities (Moukarzel et al. 2021). Colonization by a diverse AMF community can enhance grapevine growth and nutrient uptake (Moukarzel et al. 2022) and may help maintain plant development under changing environmental conditions (Wagg et al. 2011). Previous studies indicate a host preference between *Vitis* L. and AMF, with plant growth and development heavily reliant on AMF colonization. For example, 40 different AMF taxa were detected, which formed associations with grapevines and inter-row vegetation with communities differing based on the identity of the host plant, indicating that the *'Vitis'* preferentially interacts with a subgroup of the vineyard fungal community (Holland et al. 2014). Another key factor influencing the composition of the soil microbial community is vineyard management type (conventional, organic, or integrated), as the use of biocides can negatively impact soil microorganisms, including AMF (Massa et al. 2020). Integrated pest management (IPM) employs selective, less hazardous pesticides applied in smaller quantities and with lower frequency compared to conventional practices (Novello et al. 2017). Numerous studies have investigated AMF biodiversity in vineyards, focusing on both AMF present in the soil and those associated with grapevine roots (Schreiner 2005, 2007; Balestrini et al. 2010; Lumini et al. 2010; Likar et al. 2013; Holland et al. 2014).

This study proposed that the fungal microbiome of vineyards varies significantly based on a combination of factors, including geographical location, soil physicochemical properties, vineyard management practices, and the frequency of chemical sprays and fertilizers applied. The aim of our work was to: i) collect soil samples and grapevine root samples from five vineyards across different wine-growing sub-regions of the Czech Republic; ii) conduct a physical–chemical analysis of the soil samples; iii) isolate DNA from grapevine roots and analyse the associated microbiome, with a focus on the AMF community in each sub-region, using metagenomic analysis; and iv) evaluate the composition of AMF communities in relation to the specific conditions of each habitat.

## Material and methods

### Study region

Soil and grapevine root samples were collected from five vineyards located in different wine-growing sub-regions of the Czech Republic (Table 1). Sampling was conducted in the wine-growing villages of Třebívlice, Velké Pavlovice, Velké Němčice, Mikulčice, and Nový Šaldorf-Sedlešovice. The wine-growing village of Třebívlice is the only one located in the Bohemia wine region and is very distant from the remaining municipalities (approximately 300 km). The other wine-growing villages belong to the Moravia wine region and are only a few dozen kilometers apart (a maximum of 70 km, the distance between Nový Šaldorf-Sedlešovice and Mikulčice) (Figure S1). In the Morava wine region, all samples were taken from the Traminer grapevine variety grafted onto SO4 rootstock, while in the Bohemia wine region, samples were collected from the Pinot Gris variety on SO4 rootstock (Table 1). The Bohemia region is one of the colder areas where the Traminer variety is less commonly cultivated. For this reason, the Pinot gris variety, more typical of the region, was selected for sampling (although sampled rootstocks were the same in both regions). All vineyards followed the principles of IPM, including the greening of every second inter-row, and the stubble strip was mechanically cultivated. The number of fertilizer and grapevine protection product applications in the Mikulov, Slovácko, and Znojmo sub-regions is provided in Tables S1 and S2. For the Litoměřice and Velké Pavlovice sub-regions, these data were not obtained.

**Table 1:** Sampling locations and concentrations of extracted DNA

Wine sub-region	Winery, wine-growing village	Geographical coordinates	Grape variety	Rootstock	Sample identification	DNA concentration [ng.mL^−1^]
Litoměřice	Winery Johann W, Třebívlice	N 50°27.39727' E 13°53.93127'	Pinot gris	SO4	LE 1	68.79
					LE 2	66.24
					LE 3	73.32
					LE 4	25.59
Mikulov	Winery Šilinek, Pavlov	N 48°52.28142' E 16°39.98510'	Traminer	SO4	ME 1	150.84
					ME 2	107.28
					ME 3	122.75
					ME 4	65.31
Velké Pavlovice	Winery Kamil Prokeš, Velké Němčice	N 48°54.04198' E 16°49.21468'	Traminer	SO4	PE 1	67.36
					PE 2	71.61
					PE 3	112.72
					PE 4	96.66
Slovácko	Winery Dvořáček LTM s.r.o, Mikulčice	N 48°49.15787' E 17°2.91517'	Traminer	SO4	SE 1	40.28
					SE 2	155.01
					SE 3	37.99
					SE 4	28.36
Znojmo	Winery Špalek, Nový Šaldorf	N 48°49.56435' E 16°3.35207'	Traminer	SO4	ZE 1	69.48
					ZE 2	90.81
					ZE 3	59.14
					ZE 4	64.66

#### Soil and root sampling

Sampling was conducted in autumn 2020. From each vineyard, four soil samples were collected at two depths (0–30 cm and 30–60 cm) using a spade, resulting in a total of eight soil samples per vineyard. A complete soil analysis was performed on these samples. Additionally, root samples were collected from the same locations for metagenomic identification of microorganisms, with four root samples taken per vineyard. Sampling locations were conducted in a single row and included the top of the slope, two spots in the middle, and the bottom of the slope to capture a cross-section representation of the vineyard. Approximately 800 g of soil and 50 g of roots were collected from each site. All soil and root samples were stored at −20 °C prior to processing.

#### Soil chemical and physical analyses

All soil analyses were conducted at the Regional Agricultural Laboratory (ZEVOS-Agro, Zlechovská 1731, Staré Město u Uherského Hradiště, Czech Republic). The determination of exchangeable nutrients (phosphorus, potassium, calcium, magnesium) was performed using the MEHLICH III method For the analysis of zinc, manganese, and iron concentration, dried soil samples were extracted using a DTPA-TEA solution, and the filtrate was analysed by atomic absorption spectrometry (Lindsay and Norvell 1978). The sulfur concentration was measured using the filtration method according to Combs et al. (1998). The percentage of humus was quantified using the Walkley–Black method (Walkley and Black 1934). The conductivity of the soil extract was evaluated using a conductivity electrode (Vernier, Beaverton, Oregon, USA). The active lime (CaCO_3_) content was analysed by the ammonium oxalate titration method (Drouineau 1942). The total cation exchange capacity (CEC) was determined from soil filtrate using Nessler’s reagent and measured with a spectrophotometer (Pansu and Gautheyrou 2006). The final analysis of the soil test was the examination of soil grain composition, which was performed using the sedimentation method (Kettler et al. 2001).

#### DNA extraction

Grapevine root samples were used for DNA extraction. Roots were first washed with tap water, then sterilized by rinsing with ethanol and passing through a flame. Subsequently, samples were scraped with a sterile scalpel and the samples were frozen in mortars at −80 °C for 2 h. Next, the samples were homogenized with a pestle to a fine powder which was used for DNA extraction according to the protocol of the commercial NucleoSpin Tissue kit (Macherey–Nagel, Düren, Germany). The concentration of the extracted DNA samples was measured using a commercial Quant-iT™ PicoGreenTM dsDNA Assay kit (Invitrogen, Waltham, Massachusetts, USA) and a fluorimeter. The measured DNA concentration values are presented in Table 1.

#### High-throughput sequencing

The extracted DNA was used to prepare two libraries (ITS and LSU) according to the Illumina 16S Metagenomic Sequencing Library Preparation protocol (San Diego, USA).

In the case of the ITS library, the sequencing of which was aimed at defining the complete spectrum of fungal microorganisms in the samples, primers gITS7: GTG AAT CAT CGA ATC TTT G (Ihrmark et al. 2012) and ITS4: TCC TCC GCT TAT TGA TAT GC (White et al. 1990) targeting a fragment of the ITS region (~ 500–600 bp) were used for the amplification cycle. For ITS primers, a temperature program was used, which included initial denaturation at 95 °C for 10 min followed by 35 cycles of 94 °C/20 s, 47 °C/30 s, 72 °C/20 s; and final elongation at 72 °C for 7 min.

In the case of the preparation of the LSU library, which was aimed at defining the AMF spectrum in the samples, primers LR1: GCA TAT CAA TAA GCG GAG GA and FLR2: GTC GTT TAA AGC CAT TAC GTC (van Tuinen et al., 1998) were used for the amplification cycle targeting an ~ 700–750 bp LSU fragment. For LSU primers, a temperature program was used that included initial denaturation at 95 °C for 4 min followed by 34 cycles of 94 °C/1 min, 55 °C/1 min, 72 °C/1 min; and final elongation at 72 °C for 10 min.

PCR was performed in 50 μL reaction volumes, consisting of 25 μL of Q5® High-Fidelity 2 × Master Mix (NEB, Ipswich, UK), 2.5 μL of each primer (10 µM), 2 μL of template DNA, and 18 μL of nuclease-free water. The Nextera XT chemistry protocol was followed, and PCR constructs were verified as described by Špetík et al. (2022).

#### Bioinformatics and data evaluation

Sequencing data were processed using the SEED2 pipeline (Větrovský and Baldrian 2013). For first removal of low-quality sequences (reads) we used the built-in USEARCH v. 8.1.1861 (Edgar 2010). The threshold for discarded reads was set to Phred quality score = 30 and with trimming to a minimum length of 200 bases. Next, clustering at the 97% similarity cut-off, denoising and exclusion of chimeras using the built-in MAFFT v. 7.222.64 (Katoh et al. 2009) and USEARCH (Edgar 2010) algorithms was performed. The resulting clustered table was compared with the UNITE v. 9.0 database (Abarenkov et al. 2024) and classified to the taxonomic level of genus.

The OTU table with classified microorganisms and abundances was used for data processing and graph creation in MicrobiomeAnalyst (Dhariwal et al. 2017). Alpha diversity was expressed using three Hill numbers: richness = number of species (^0^D), the Shannon´s diversity (^1^D), and the Simpson´s diversity (^2^D). The formulas for calculating Hill numbers are provided in Table S3. Beta diversity analysis was performed in MicrobiomeAnalyst. Beta diversity analysis was performed using the PCoA method using the Bray–Curtis Index. Good’s coverage values and rarefaction curves also were calculated (Figure S5). Sub-region and soil type were used separately as experimental factors. We set the threshold of statistical significance for both analyses at p = 0.01. Venn diagrams were created at http://bioinformatics.psb.ugent.be/webtools/Venn/. Linear discriminant analysis (LDA) effect size (LEfSe) was generated using the Galaxy web application (Afgan et al. 2022) available at: http://huttenhower.sph.harvard.edu/galaxy/. LEfSe was used to identify the most diverse taxa among sub-regions. The cladogram generated by LEfSe illustrates fungal differences at the phylum, class, family, and genus levels among the sub-regions (relative abundance ≤ 0.5%; p = 0.01). Each successive circle represents a phylogenetic level, while coloured regions indicate taxa enriched in different sub-regions. A bar graph from LEfSe presents LDA scores for fungi, with only taxa meeting a significant LDA threshold > 2 included. Furthermore, the ecological function of individual microorganisms in FUNGuild v 1.0 (available at: http://www.funguild.org/) was determined (Nguyen et al. 2016). Guilds were classified according to three trophic modes: pathotrophs, saprotrophs and symbiotrophs. All OTUs that did not match taxa in the database were classified as “unassigned”. To achieve homogeneity in the distribution of results, the number of taxa falling into each category was converted into percentages. For the statistical evaluation of the combined effect of trophic mode and sub-region, a two-way analysis of variance (ANOVA, significance level p = 0.01) was used, conducted in the Statistica 14 CZ (StatSoft, Prague, Czech Republic) software. Subsequently, the least significant difference (LSD) post-hoc test with a significance level of p = 0.01 was used. The results of the FUNGuild analysis were graphically processed in Microsoft Excel 365 (Microsoft, Redmond, Washington, USA). In the Cytoscape 3.10.0 software (Shannon et al. 2003) with the CoNet plugin (Faust and Raes 2016), interaction networks among the individual microorganisms were created (only genera with an abundance greater than 100 were included in this analysis). For each sub-region and each library, one network was created that displays the fungal genera present (nodes) and the interactions among them (edges). The colour of the edges distinguishes between positive (green) and negative (red) interactions.

## Results

### Soil chemical and physical analyses

The results of the physicochemical analyses of the soils are presented in Table 2. The sub-region that differed the most from other sub-regions was Slovácko, where low soil pH, low levels of phosphorus (P), zinc (Zn), manganese (Mn), iron (Fe), and humus, as well as a low CEC value, were observed. In other sub-regions, soil pH was high, except for the Mikulov sub-region, where it was optimal. Phosphorus and humus content were optimal in all sub-regions except for Slovácko. The levels of Zn, Mn, and Fe were low in all sub-regions, except for the Litoměřice sub-region, where Zn had an optimal value. The CEC value, as for Slovácko, was low in all other sub-regions, reaching optimal values only in the Mikulov sub-region. Other elements (potassium, sulfur, active lime) and conductivity were at optimal levels across all sub-regions. Calcium concentration was high in all sub-regions except for Slovácko, where it was optimal. Magnesium concentration was high in the Mikulov and Velké Pavlovice sub-regions, while values in other sub-regions were optimal. The soil type was determined from the percentage of sand, clay, and silt in the samples. In the Litoměřice and Velké Pavlovice sub-regions, there was clay; in Mikulov and Znojmo, there was clay loam; and in Slovácko, there was sandy loam.

**Table 2:**
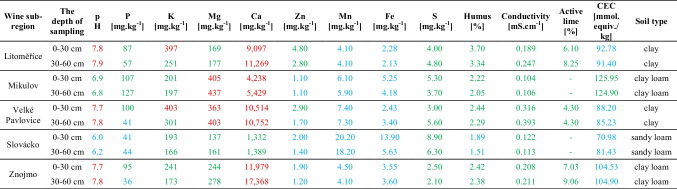
Soil Chemical and Physical Analyses: font color defines low values (blue), optimal values (green) and high values (red)

Note: Extractants used for determining the content of individual elements and soil properties: P, K, Ca, Mg—MEHLICH III; Zn, Mn, Fe—DTPA-TEA solution; S—barium chloride (BaCl₂); Humus—Mohr's salt solution; active lime—potassium permanganate (KMnO₄)

#### High-throughput sequencing

All obtained raw sequences are available in the NCBI database under BioProject PRJNA1007931, which includes two BioSamples: SAMN37105855 for the ITS library and SAMN37105857 for the LSU library. For the ITS library, in the first amplification cycle, 500–600 bp products were visible on the agarose gel after electrophoresis in all 20 samples, which were subsequently purified and further analysed. For the LSU library, in the first amplification cycle, products sized 700–750 bp were visible on the agarose gel after electrophoresis in all four samples from the Litoměřice and Mikulov sub-regions, as well as in three samples from the Velké Pavlovice and Znojmo sub-regions. Amplification did not occur in the samples from the Slovácko sub-region; therefore, those samples were not analysed further. The lack of amplification in these samples was likely due to the low concentration of AMF. A total of 14 LSU samples were subsequently purified and further analysed.

Sequencing the ITS library yielded 2,347,485 sequences with an average Phred quality score 37.81. After processing the sequences, OTU tables were filtered, with sequences not classified up to the genus taxonomic category being removed. The final OTU table for ITS libraries containing 109 fungal genera (OTUs) was created (Table S4). Sequencing the LSU library yielded 11,357,594 sequences with an average Phred quality score 36.32. In the resulting Table 186 OTUs were included (Table S5). In all sub-regions (ITS library), the phyla *Ascomycetes* and *Basidiomycetes* were the most numerous. In the Litoměřice, Mikulov, Velké Pavlovice, and Znojmo sub-regions, *Ascomycetes* prevailed (74.91%, 77.82%, 92.15%, and 70.21%, respectively). *Basidiomycetes* in these sub-regions were represented by 18.77% for Litoměřice, 12.92% for Mikulov, 7.11% for Velké Pavlovice, and 29.49% for Znojmo. In the Slovácko sub-region, the phylum *Basidiomycetes* predominated (57.47%), while *Ascomycetes* were represented by 39.80%. The phylum *Glomeromycetes*, which includes AMF, was represented by only 4.89% for Litoměřice, 5.66% for Mikulov, 0.08% for Velké Pavlovice, 2.05% for Slovácko, and 0.07% for Znojmo. On the other hand, in the LSU library the most represented phylum in all sub-regions was *Glomeromycetes*, represented by 98.71% for Litoměřice, 94.44% for Mikulov, 83.77% for Velké Pavlovice, and 97.83% for Znojmo.

**Fig. 1:**
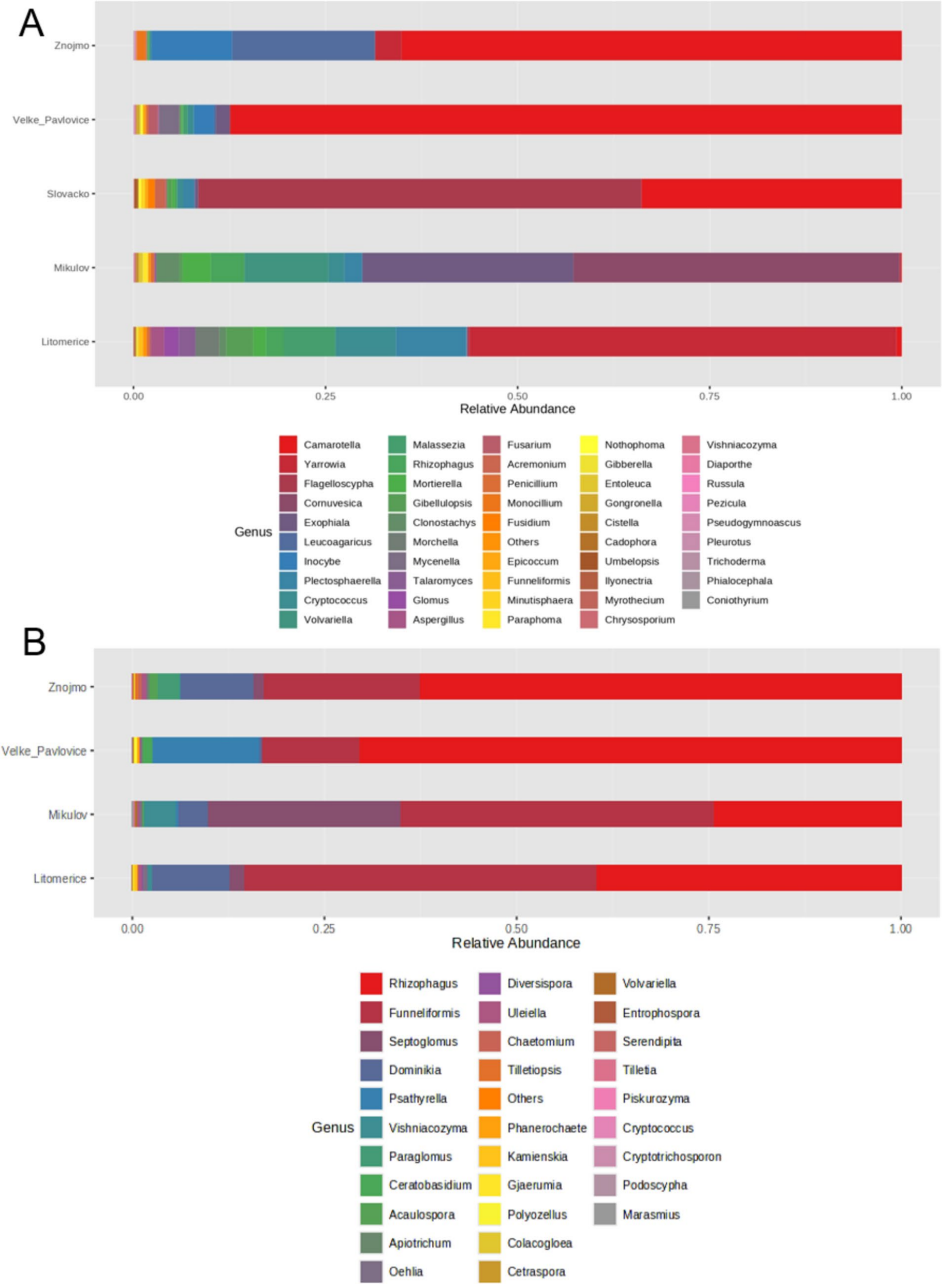
Percentage representation of the most abundant OTUs in the ITS library (**A**) and in the LSU library (**B**). In the case of the LSU library, the Slovácko sub-region (**B**) was not evaluated because of low amplification with LS primers

From ITS library results, in the Litoměřice sub-region, the most represented genus was *Yarrowia* (55.58%), in the Mikulov sub-region *Cornuvesica* (42.42%), in the Slovácko sub-region *Flagelloscypha* (57.68%), and in the Velké Pavlovice and Znojmo sub-regions *Camarotella* (87.34% and 65.11%, respectively) (Fig. 1A). The results from the LSU library indicate that the most represented genus in the sub-region was either *Funneliformis* (46.01% in Litoměřice and 41.05% in Mikulov) or *Rhizophagus* (70.32% in Velké Pavlovice and 62.50% in Znojmo) (Fig. 1B).

**Fig.2:**
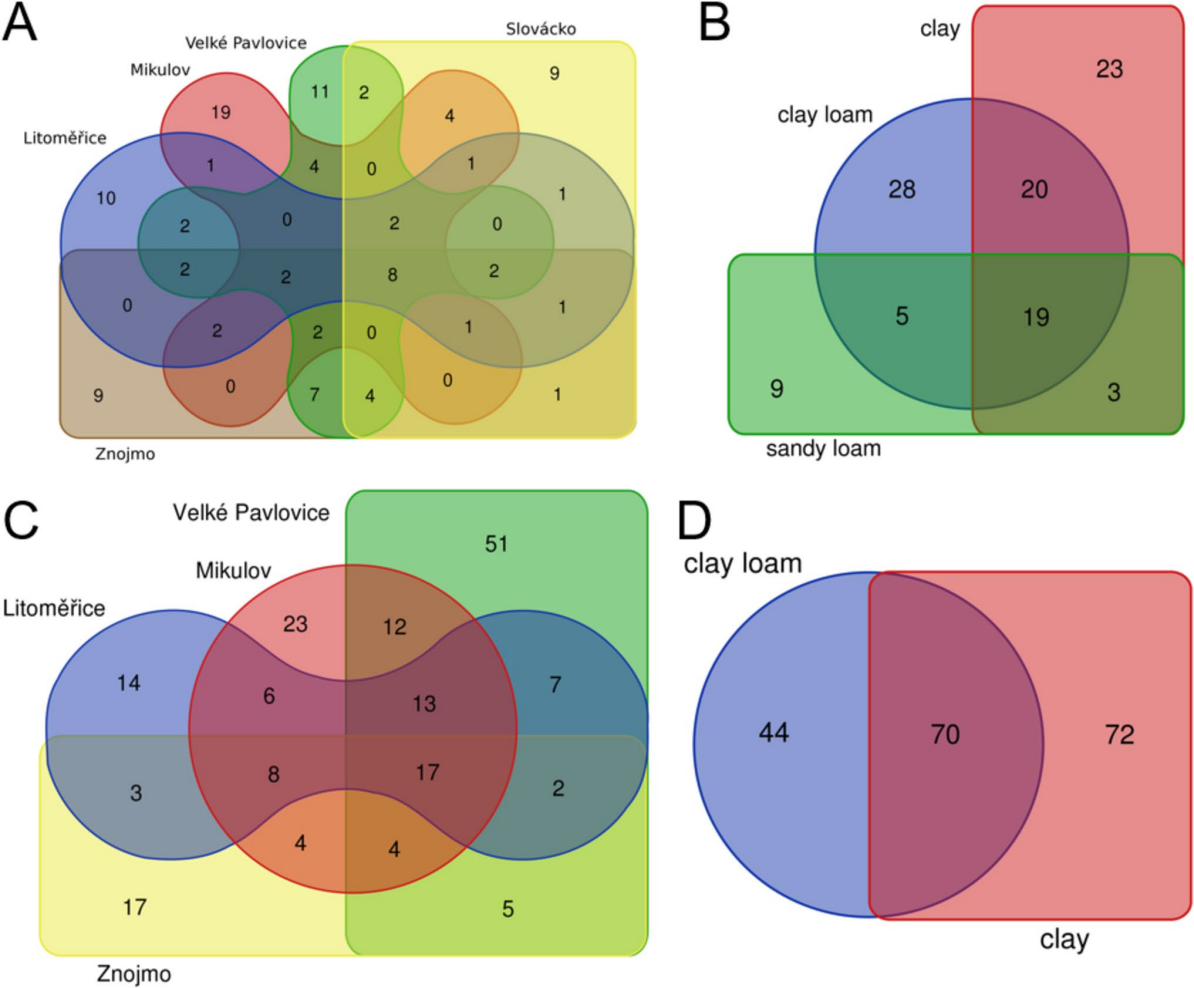
Venn diagrams of shared OTUs across sub-regions and type of soil for the ITS library (**A**, **B**) and LSU library (**C**, **D**). In the case of the LSU library, the Slovácko sub-region (**C**) and sandy soil (**D**) were not evaluated because of low amplification with LSU primers

In the ITS library all sub-regions shared 8 OTUs. The sub-regions of Mikulov (19), Velké Pavlovice (11), and Litoměřice (10) had the most unique OTUs, while Slovácko and Znojmo (9) had the fewest (Fig. 2A). In terms of soil type, all types (clay, clay loam, sandy loam) shared 19 OTUs. The soil types of clay loam (28) and clay (23) displayed the most unique OTUs, while sandy loam had 9 unique OTUs (Fig. 2B). In the LSU library all sub-regions shared 17 OTUs. The sub-regions of Velké Pavlovice (51) and Mikulov (23) had the most unique OTUs, while Litoměřice (14) had the fewest (Fig. 2C). In terms of soil type, all types (clay and clay loam) shared 70 OTUs. The soil type clay loam had 44 unique OTUs, while clay had 72 unique OTUs (Fig. 2D).

For ITS libraries, the highest alpha diversity was observed in the Velké Pavlovice sub-region (^0^D = 50; ^1^D = 2.03; ^2^D = 1.32) and the lowest in the Litoměřice and Mikulov sub-regions (^0^D = 35; ^1^D = 8.85; ^2^D = 4.00 and ^0^D = 46; ^1^D = 7.10; ^2^D = 4.17, respectively). For LSU libraries, in terms of the experimental factor "sub-region," the highest alpha diversity was also observed in the Velké Pavlovice sub-region (^0^D = 111; ^1^D = 2.63; ^2^D = 1.89). For the other sub-regions (except for the Slovácko sub-region, which was not analysed because of its low amplification with LSU primers) similar values of ^1^D and ^2^D were observed, indicating that alpha diversity among sub-regions was more balanced for LSU libraries compared to ITS libraries. In terms of the experimental factor "soil type," similar levels of alpha diversity were observed across all soil types in both the ITS and LSU libraries. Sandy soil in LSU libraries was not analysed because, as mentioned above, amplification using LSU primers did not occur in the Slovácko sub-region which had that soil type (Table 3).

**Table 3:** Calculation of alpha diversity values: N/A – Slovácko, and consequently sandy soil, were not analysed because of low amplification with LSU primers in these samples

Factor	Primers	Wine sub-region	Richness (S; ^0^D)	Shannon index (H´)	Simpson index (D)	Gini-simpson index (GS)	Shannon´s eveness (J)	Simpson´s eveness (equitability)	Shannon´s diversity (^1^D)	Simpson´s diversity (^2^D)
Wine sub-region	ITS	Litoměřice	35	2.18	0.25	0.75	0.61	0.11	8.85	4.00
		Mikulov	46	1.96	0.24	0.76	0.51	0.09	7.10	4.17
		Velké Pavlovice	50	0.71	0.76	0.24	0.18	0.03	2.03	1.32
		Slovácko	37	1.24	0.42	0.58	0.34	0.06	3.46	2.38
		Znojmo	43	1.09	0.47	0.53	0.29	0.05	2.97	2.13
	LSU	Litoměřice	70	1.18	0.38	0.62	0.28	0.04	3.26	2.64
		Mikulov	87	1.45	0.29	0.71	0.33	0.04	4.28	3.44
		Velké Pavlovice	111	0.97	0.53	0.47	0.21	0.02	2.63	1.89
		Slovácko	N/A	N/A	N/A	N/A	N/A	N/A	N/A	N/A
		Znojmo	60	1.19	0.44	0.56	0.29	0.04	3.29	2.26
Soil type	ITS	clay	67	0.92	0.30	0.70	0.22	0.05	2.51	3.33
		clay loam	74	1.48	0.38	0.62	0.34	0.04	4.39	2.63
		sandy loam	37	1.24	0.42	0.58	0.34	0.06	3.46	2.38
	LSU	clay	142	1.26	0.38	0.62	0.26	0.02	3.53	2.66
		clay loam	114	1.50	0.30	0.70	0.32	0.03	4.50	3.37
		sandy loam	N/A	N/A	N/A	N/A	N/A	N/A	N/A	N/A

**Fig. 3:**
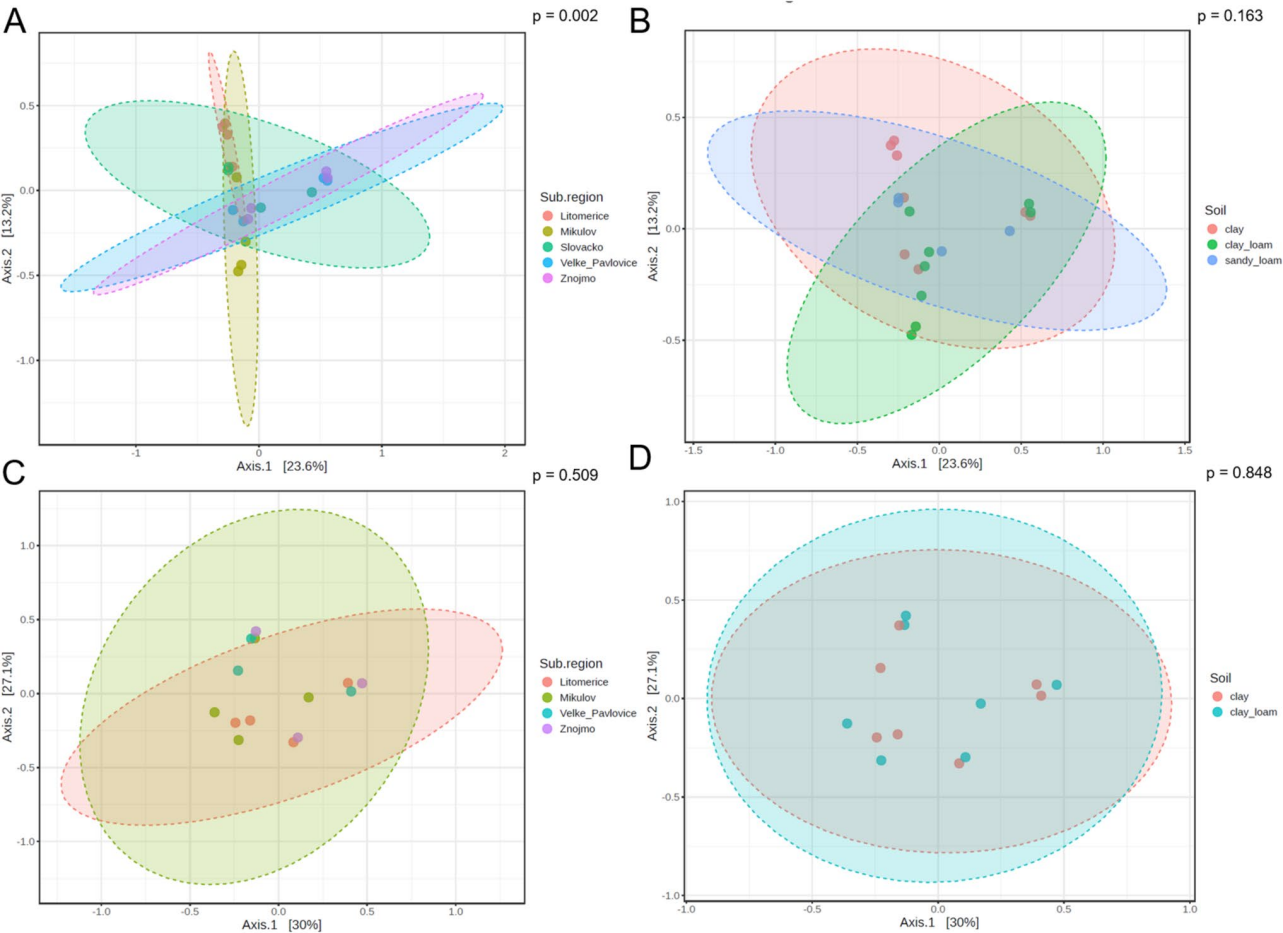
Beta diversity for the ITS library: sub-region (**A**) and type of soil (**B**); and the LSU library: sub-region (**C**) and type of soil (**D**). The ellipses represent the 95% confidence interval assuming a normal distribution. The colour of the points and ellipses corresponds to the experimental factor (sub-region or soil type). In the case of the LSU library, the Slovácko sub-region (**C**) and sandy soil (**D**) were not evaluated because of low amplification with LSU prime

From the results of beta diversity for the ITS library, the experimental factor 'sub-region' (*p* = 0.002) was statistically significant, while the experimental factor 'soil type' (*p* = 0.163) was statistically insignificant. This means that diversity was influenced by sub-region but not by soil type. The sub-region of Slovácko was the most distinct from the others. Furthermore, the sub-regions formed two groups with similar diversity: i) Litoměřice and Mikulov; ii) Velké Pavlovice and Znojmo (Fig. 3A). Individual soil types did not differ significantly from each other (Fig. 3B). From the beta diversity results for the LSU library, both sub-regions (*p* = 0.509; Fig. 3C) and soil type (*p* = 0.848; Fig. 3D) did not have a statistically significant effect on the diversity of microorganisms.

LEfSe for the ITS library detected 41 fungal clades in soil samples, distinguishing microbial communities among sub-regions. The Mikulov sub-region had a higher number of variously abundant fungal clades (11) than the other sub-regions: 10 in Znojmo, 9 in Litoměřice, 8 in Slovácko, and 3 in Velké Pavlovice (Figure S2A). LEfSe analysis for the LSU library detected 27 fungal clades in root samples, distinguishing microbial communities among sub-regions. The Velké Pavlovice sub-region had a higher number of variously abundant fungal clades (17) than the other sub-regions (9 in Znojmo and 1 in Litoměřice). When the parameters were set to p = 0.01 and LDA threshold > 2, the Mikulov sub-region did not exhibit any variously abundant fungal clades (Figure S2B).

Figure S3 depicts the interaction networks among fungal genera for each sub-region in the ITS library. In the Mikulov sub-region, the ratio of negative to positive interactions was almost equal, with 54% of interactions being positive and 46% negative. In the other sub-regions, negative interactions among microorganisms prevailed, ranging from 64 to 91% (Table S6). Figure S4 displays the networks of interactions among fungal genera for each sub-region in the LSU library, except for the Slovácko sub-region, where AMF were not detected. In the Mikulov sub-region, the number of positive interactions slightly prevailed, with 58% of positive interactions and 42% of negative interactions detected. In other sub-regions, positive interactions prevailed among fungal genera, ranging from 64 to 97% (Table S7).

**Fig. 4:**
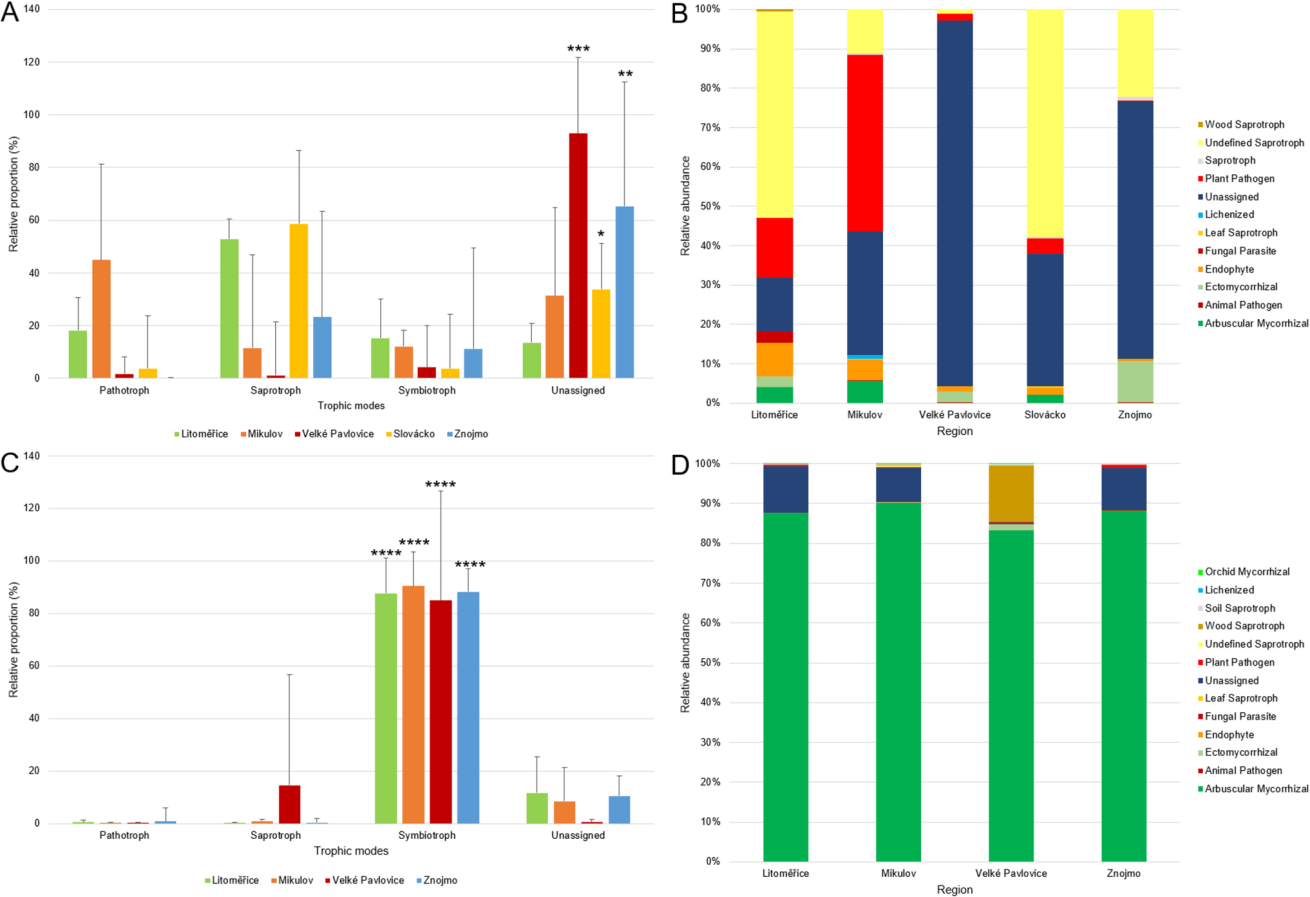
Variations in fungal function and composition of fungal functional groups (guilds) inferred by FUNGuild for the ITS library (**A**, **B**) and LSU library (**C**, **D**). Error bars represent + 1 standard deviation. Statistically significant values according to the two-way ANOVA and LSD test are marked with asterisks. In the ITS library, statistically significant differences were found for the Unassigned and the Slovácko sub-region, which differed only within the Unassigned trophic mode from the Velké Pavlovice and Znojmo sub-regions (*). Furthermore, within the Unassigned group, the Znojmo sub-region differed from all trophic modes and sub-regions except for the Saprotroph trophic mode in the Slovácko and Znojmo sub-regions (**). The last observed significant difference was for the Unassigned trophic mode in the Velké Pavlovice sub-region, which differed from all trophic modes and sub-regions except for Unassigned in the Znojmo sub-region (***). In the LSU library, all sub-regions (Litoměřice, Mikulov, Velké Pavlovice, Znojmo) in the Symbiotroph trophic mode differed from all other trophic modes and sub-regions. Within the Symbiotroph, no statistically significant difference among sub-regions was observed (****)

For the ITS library, 86.35%, 68.64%, 7.03%, 66.22%, and 34.64% of OTUs from the sub-regions of Litoměřice, Mikulov, Velké Pavlovice, Slovácko, and Znojmo were identified as trophic modes including saprotrophs, symbiotrophs, and pathotrophs, while the remaining OTUs were not assigned (Fig. 4A). The most represented functional group (Fig. 4B) in the Litoměřice sub-region was Undefined Saprotroph (52.36%), in the Mikulov sub-region Plant Pathogen (44.82%), in the Velké Pavlovice sub-region Unassigned (92.97%), in the Slovácko sub-region Undefined Saprotroph (57.71%), and in the Znojmo sub-region Unassigned (65.36%). For the LSU library, 88.33%, 91.48%, 99.54%, and 89.43% of OTUs from the sub-regions of Litoměřice, Mikulov, Velké Pavlovice, and Znojmo were identified as trophic modes including saprotrophs, symbiotrophs, and pathotrophs, while the remaining OTUs were not assigned (Fig. 4C). The most represented functional group (Fig. 4D) in all sub-regions was Arbuscular Mycorrhizal, with values of 87.65%, 90.16%, 83.37%, and 88.24% for the Litoměřice, Mikulov, Velké Pavlovice, and Znojmo sub-regions, respectively.

#### Comparison of individual analyses

Within the ITS library, the most abundant species was *Camarotella*, which prevailed especially in the sub-regions of Velké Pavlovice and Znojmo, where high soil pH (7.7–7.8) and elevated Ca concentrations (10,514–17,368 mg.kg^−1^) were measured. In the case of the LSU libraries, *Rhizophagus* and *Funneliformis* species predominated in all sub-regions.

The high sensitivity of LSU primers to AMF was demonstrated. For comparison, 46 ITS sequences and 1,690 LSU sequences were detected for the genus *Entrophospora*; for the genus *Funneliformis*, there were 11 ITS sequences and 1,609,998 LSU sequences; for the genus *Paraglomus*, 269 ITS sequences and 30,192 LSU sequences; for the genus *Rhizophagus*, 282 ITS sequences and 2,184,375 LSU sequences; and for the genus *Septoglomus*, 8 ITS sequences and 367,460 LSU sequences. Other genera belonging to the AMF group according to FunGuild (*Acaulospora, Ambispora, Archaeospora, Cetraspora, Corymbiglomus, Dentiscutata, Diversispora, Fuscutata, Gigaspora, Otospora, Redeckera, Sacculospora, Scutellospora*) were detected only in the LSU library.

The largest number of identified ITS sequences (37,986) was in the Velké Pavlovice sub-region. In the Znojmo sub-region, 35,486 ITS sequences were identified, in the Slovácko sub-region 15,433 ITS sequences, in the Mikulov sub-region 3,855 ITS sequences, and in the Litoměřice sub-region 1,678 ITS sequences. The largest number of identified LSU sequences (1,635,006) was in the Litoměřice sub-region. In the Mikulov sub-region, 1,314,765 LSU sequences were identified; 796,241 LSU sequences in the Velké Pavlovice sub-region; and 1,059,961 LSU sequences in the Znojmo sub-region. In the samples from the Slovácko sub-region, there was no amplification with LSU primers due to the low concentration of AMF in the samples.

When comparing the interaction networks, negative interactions predominate in the networks for the ITS library, while positive interactions prevail in the networks for the LSU library among the most represented fungal genera. The exception is the networks for the Mikulov sub-region, where the ratio of negative to positive interactions was almost equal in both cases.

According to FunGuild, 87.65%, 90.16%, 83.37%, and 88.24% of LSU sequences in the sub-regions of Litoměřice, Mikulov, Velké Pavlovice, and Znojmo fell into the AMF group, respectively. In all these sub-regions, the Ca concentration was elevated, which did not negatively affect the AMF community. Additionally, the optimal humus content probably had a positive effect on AMF in all these sub-regions. In all these sub-regions, there was sufficient phosphorus for grapevine in the upper soil layer (0–30 cm, Table 2). AMF also positively influenced the suppression of the fungal pathogen *Cadophora*, with 32 ITS sequences of this genus detected in the Slovácko sub-region, 2 ITS sequences in the Litoměřice and Znojmo sub-regions, and 0 ITS sequences in the Mikulov and Velké Pavlovice sub-regions. Furthermore, a reduction in the occurrence of the root rot pathogen *Roesleria* also was observed, with 20 ITS sequences detected in the Slovácko sub-region and 0 ITS sequences in the other sub-regions.

## Discussion

This study characterized the fungal microbiomes of the endophytic environment and AMF colonization across five wine sub-regions in the Czech Republic. Soil microbial communities play a critical role in grape cultivation, as they act as reservoirs for grape-associated microorganisms (Zarraonaindia et al. 2015), influencing the development of region-specific wine characteristics (Bokulich et al. 2016).

Geographic location was confirmed to significantly influence the composition of the fungal microbiome (ITS library), with only 8 OTUs shared across all sub-regions, a finding consistent with other studies. For instance, Coller et al. (2019) analysed soil samples from 10 vineyards across 4 wine regions in Italy’s Trentino province, identifying 12,101 OTUs, of which only 5 were shared among all vineyards. Similarly, Gobbi et al. (2022) analysed soil samples from 200 sites across 13 countries on 4 continents (Europe, Africa, America, and Australia) and identified 909 OTUs, with just 24 shared among all continents.

The ITS library especially captures competitive interactions because it includes pathogenic and saprotrophic fungi that often compete for resources. In contrast, the LSU library preferentially detects mutualistic mycorrhizal fungi, which form positive interactions with host plants and other microorganisms. The Mikulov sub-region stands out with a balanced ratio of interactions, likely because of stable ecological conditions or different soil management practices. This result highlights how different molecular markers can influence the interpretation of ecological networks and underscores the importance of using multiple approaches to understand fungal dynamics in vineyards.

Sequencing ITS libraries and creating a Venn diagram reveals a comparable number of unique OTUs for clay and clay loam (23 and 28, respectively). In the LSU library, these soil types are significantly different from each other, with 72 unique OTUs found in clay and 44 specific OTUs in clay loam. In all sub-regions, the predominant fungal phyla in the ITS libraries were *Ascomycetes* and *Basidiomycetes*, consistent with findings from other studies. Martínez-Diz et al. (2019) analysed samples from five young vineyards in La Rioja, Spain, where *Ascomycetes* (50.2% to 60.6%, depending on the site) and *Basidiomycetes* (15.0% to 20.9%) made up nearly 70% of the total fungi detected. In our study, *Ascomycetes* ranged from 39.8% to 92.2%, and *Basidiomycetes* from 7.1% to 57.5%, jointly representing 99% of the total ITS fungi. Similarly, Coller et al. (2019) found that in 10 Italian vineyards, the dominant phyla were *Ascomycetes* (51.8%), *Zygomycetes* (20.1%), and *Basidiomycetes* (11.2%). In our study, *Ascomycetes* accounted for 75% and *Basidiomycetes* for 25%, with *Zygomycetes* not detected. Gupta et al. (2019) observed that *Ascomycetes* and *Basidiomycetes* were the dominant phyla at two soil depths in an Australian vineyard, representing 84% of identified species. In our results, these two phyla accounted for 99%, 15% higher than in Gupta et al. (2019).

Our research confirmed that LSU primers are more sensitive than ITS primers for AMF detection. The representation of the phylum *Glomeromycetes* ranged from 0.1% to 5.7% in the ITS library, compared to 83.7% to 98.7% in the LSU library. Aguilar et al. (2020) found similarly low *Glomeromycetes* representation (2–3%) employing ITS sequencing in Argentine vineyards, consistent with our findings. Řezáčová et al. (2016) analyzed AMF communities using both LSU and ITS primers, reporting that 71.9% of LSU sequences were identified as *Glomeromycetes*, compared to only 21.7% of ITS sequences. In our study, *Glomeromycetes* composed up to 5.7% of ITS sequences and up to 98.7% of LSU sequences. Similarly, Gupta et al. (2019) found that *Glomeromycetes* detected by ITS primers accounted for less than 0.5% of fungi in Australian vineyard soils, comparable to the 0.1% found in the Znojmo sub-region.

The AMF community was found to be influenced by soil type and nutritional status. Only trace amounts of AMF were detected in the Slovácko sub-region. This may be due to the soil type (the only sub-region where sandy loam was detected) and the lower soil pH compared to other sub-regions. Furthermore, low values of phosphorus, zinc, manganese, iron, humus, and CEC were also recorded, likely because no fertilizer had been applied to the soil in the three years prior to sampling. Aliasgharzad et al. (2010) confirmed that AMF are generally more abundant in deeper soil layers (20–30 cm), especially when the pH is above 6.5 and organic matter content is around 2%. In mineral soil, the less than adequate foliar Mn concentration might have reflected retarded root growth because of soil density, low pH, and relatively high exchangeable Al (Parsons and Uren 2007). We detected a low abundance of AMF in the Slovácko sub-region, where the soil is sandy, the pH is 6, the highest Mn content 18.2 to 20.2 mg.kg^−1^ from the evaluated soils, and the humus content is 1.89%. Excessive phosphorus (P) availability for plants reduces the need for interaction with arbuscular mycorrhizal fungi (AMF) (Grant et al. 2005; Zuccarini 2010). High alkalinity (pH > 9) can negatively impact the formation and propagation of arbuscular mycelium (Zuccarini 2010). However, the ability of AMF to adapt to alkaline environments depends on the fungus species and the plant with which AMF establish a symbiotic relationship. Parihar et al. (2019) concluded that species from the *Glomeraceae* were the most frequently reported under high alkalinity (pH 7.7 to 9.4) and species such as *Rhizophagus fasciculatus* under strong alkaline condition.

Optimal phosphorus (P) concentration was observed in all soils with high AMF colonization (Litoměřice, Mikulov, Velké Pavlovice, Znojmo). In contrast, P deficiency was only detected in the Slovácko sub-region, where the soil pH was low. This aligns with findings by Menge et al. (1983), who reported that P deficiency occurs predominantly in acidic soils that promote phosphorus fixation. Khalil (2013) also noted that high root or leaf P levels in grapevines were associated with abundant AMF presence, consistent with the correlation between low P and low AMF concentrations in the Slovácko sub-region. Schreiner (2005) further suggested that AMF colonization and P uptake in grapevines could decrease in soils with pH values between 5 and 5.5. Although the pH in Slovácko was 6, it suggests that P intake may be affected by soil acidity.

Low iron (Fe) concentration and high calcium (Ca) concentration were observed in all soils from the sub-regions of Litoměřice, Mikulov, Velké Pavlovice, and Znojmo. However, the grapevines in these vineyards were grown on SO4 rootstock, which is known for its tolerance to elevated soil Ca levels. According to Bavaresco and Poni (2003), the SO4 rootstock can tolerate up to 17% active lime in the soil. In this study, none of the sub-regions exceeded this threshold, with the highest value recorded in the Znojmo sub-region at 9.1%.

The occurrence of AMF in the Slovácko sub-region may have been affected by the frequent application of fungicides, mainly against powdery and downy mildew. In the three years before sampling, chemical preparations were applied to the vineyard 49 times. For comparison, preparations were applied 47 times in the Znojmo sub-region; of these, 28 applications were biopreparations such as fennel oil or *Bacillus subtilis* spores. In the Mikulov sub-region, the number of applications of chemical preparations was much lower, at 26. Komárek et al. (2010) reported that long-term fungicide use, along with runoff from treated plants, leads to significant heavy metal accumulation, reaching toxic levels in vineyard surface soils. Ninkov et al. (2012) highlighted that heavy metal buildup negatively affects soil flora and fauna, potentially causing phytotoxicity in acidic soils, leaf oxidative stress, yield losses, and poorer wine quality. Meier et al. (2011) isolated metal-resistant AMF from polluted soils and noted that these populations are better adapted to metal toxicity than AMF from uncontaminated soils. Additionally, Trouvelot et al. (2015) suggested that AMF inoculation offers promise for improving plant tolerance to environmental stress in metal-contaminated soils. This approach may be particularly useful for phytoremediation in vineyards contaminated with heavy metals such as copper (Cu).

In total, results suggest that copper-based fungicide application in vineyards may decrease AMF abundance especially as in Slovácko. In this sub-region heavy application of fungicides was used, the soil parameters (such as pH, P, Zn, Mn, and Fe) were very low (Table 2), and humus content also was low. Slovácko had low abundance of almost all fungus species but interestingly *Flagelloscypha* was in two sampling points in a very high abundance, while in all other sites it was 0 or very low abundance. This species typically is found on decaying wood (Reid et al. 1964), so some plants were probably already decaying in the Slovácko sub-region. Similarly, *Camarotella*, a wood-decaying fungus genus, was detected in high abundance in the Slovácko, Velké Pavlovice, and Znojmo sub-regions. However, unlike the Slovácko sub-region, the Velké Pavlovice and Znojmo sub-regions exhibited a high abundance of mycorrhizal species.

The presence of AMF also was associated with reduced fungal pathogens such as *Cadophora* and *Roesleria*, which were present in the Slovácko sub-region but found in very low concentrations in the other sub-regions. According to Cameron et al. (2013), plants increase their defence capacity following AMF colonization, a phenomenon known as "mycorrhizal-induced resistance" (MIR). This systemic protection can shield plants from a wide range of biotrophic and necrotrophic pathogens, nematodes, and herbivorous arthropods. Moreover, Moukarzel et al. (2022) demonstrated that well-colonized AMF grapevine roots are resilient to Black foot disease caused by *Dactylonectria*/*Ilyonectria* spp. The absence of AMF in the Slovácko sub-region may explain the elevated presence of *Cadophora* and *Roesleria* there.

## Conclusion

We analyzed the fungal community and AMF colonization in five wine regions of the Czech Republic. Our findings demonstrate that microbiome composition and AMF colonization are significantly influenced by geographic location, and physicochemical attributes of the soil. Additionally, AMF were negatively affected by the frequency of fungicide applications used to protect the vines. Conversely, AMF had a positive effect on soil phosphorus levels and was associated with a reduction in fungal pathogens, though causality cannot be inferred. These results suggest that winegrowers should prioritize maintaining soil nutritional balance and reducing the frequency of fungicide applications in vineyards.

## Supplementary Information

Below is the link to the electronic supplementary material.

Supplementary file1 (XLSX 5257 KB)

## Data Availability

No datasets were generated or analysed during the current study.
